# Changes Observed in Potential Key Candidate Genes of Peripheral Immunity Induced by Tai Chi among Patients with Parkinson’s Disease

**DOI:** 10.3390/genes13101863

**Published:** 2022-10-15

**Authors:** Guang Yang, Qun Dong, Huixin Yang, Fan Wang, Linwei Chen, Junze Tang, Guoyuan Huang, Ying Zhao

**Affiliations:** 1 Physical Education Department, Shanghai Jiao Tong University, Shanghai 200042, China; 2 Department of Bioinformatics and Biostatistics, School of Life Sciences and Biotechnology, Shanghai Jiao Tong University, Shanghai 200240, China; 3 Institute of Nation Traditional Sports, Harbin Sport University, Harbin 150006, China; 4 Pott College of Science, Engineering and Education, University of Southern Indiana, Indiana, IN 47712, USA

**Keywords:** Tai Chi, Parkinson’s disease, peripheral inflammation, WGCNA, DEGs

## Abstract

Parkinson’s disease (PD) is a common progressive neurodegenerative disease characterized by motor dysfunction. Although the inhibition of inflammation by Tai Chi has been demonstrated to involve a peripheral cytokine response and may play an important role in improving the motor function of PD patients, the related specific molecular mechanisms of the peripheral immune response to Tai Chi are not fully understood. The microarray dataset ‘GSE124676’ for the peripheral immune response to Tai Chi of PD patients was downloaded from the Gene Expression Omnibus database. Differentially expressed genes (DEGs) were screened and analyzed using weighted gene co-expression network analysis (WGCNA). A total of 136 DEGs were found in the PD patients after Tai Chi, suggesting an effect of Tai Chi on the peripheral immunity of PD patients. The DEGs are mainly involved in neutrophil activation, T-cell activation, and NOD-like receptor and IL-17 signaling pathways. Furthermore, six key candidate genes (FOS, FOSB, JUNB, ZFP36, CAMP and LCN2) that are involved in peripheral inflammation and the inhibition of inflammation induced by Tai Chi were observed. The results in the present study could be conducive to comprehensively understanding the molecular mechanism involved in the effect of Tai Chi on peripheral inflammation in PD patients and providing novel targets for future advanced research.

## 1. Introduction

Parkinson’s disease (PD) is one of the most common progressive neurological degenerative disorders. PD is characterized by motor dysfunctions that are associated with such signs and symptoms as resting tremor, difficulty in initiating movements (akinesia), slowness of movements (bradykinesia), rigidity, gait disturbance and postural instability; notably, non-motor dysfunctions have been emphasized more recently [1]. It has been estimated that PD currently affects more than 4 million people aged 50 years and older worldwide, but this figure is expected to double by 2030 [2]. The management of PD is important but complex and needs to be individualized. In the early stage, the pharmacologic treatments for PD motor symptoms are primarily dopamine based, such as using levodopa preparations, dopamine agonists, and monoamine oxidase-B (MAO-B) inhibitors [3]. However, with the progress of the disease and wearing off the medication, there are long-term side effects such as symptom fluctuation, significant motor complications and cognitive dysfunction [3,4], which have become a great challenge for clinicians and neurologists. Advanced therapies for Parkinson’s disease are emerging, including functional surgery such as deep brain stimulation. Deep brain stimulation helps to improve static postural balance stability in patients with PD [5], but it requires stricter indications and a detailed assessment of the PD patient’s symptoms and dysfunction, to predict and determine the efficacy of the surgery in improving motor symptoms. Overall, there is a great need to develop therapies that are more effective but that have fewer side effects, which will be clinically meaningful for managing Parkinson’s disease.

Due to the long course and slow progression of PD and the limitations of drug/surgical therapy in relieving symptoms, alternative approaches are being advanced. Exercise interventions have been shown to alleviate PD-related symptoms [6,7]. It has been becoming an integral part of PD management because exercise may benefit PD patients in different aspects, including overall cognition, quality of life, executive control, balance and gait [8,9]. Tai Chi is a gentle, slow and mind–body harmony exercise. It has been reported to reduce the incidence of falls in older populations [10]. Tai Chi may ameliorate both the motor dysfunction and non-motor complications of PD. Two pilot studies addressed the efficacy of Tai Chi in improving postural stability in PD [11,12]. A randomized controlled trial by Li et al. showed improvements in maximal excursion, direction control, gait velocity and quality of life in PD patients after 6 months of Tai Chi exercise [13]. Interestingly, a recent study reported that, after Tai Chi training, the reduced peripheral inflammation was positively related to improved Berg balance scale (BBS) scores in PD patients [14]. All these effects from Tai Chi exercise are beneficial for the treatment and rehabilitation of PD patients, making Tai Chi a potentially important therapy for PD patients.

Peripheral inflammation could contribute to the etiology and progression of PD [15]. The results from meta-analyses revealed higher concentrations of peripheral inflammatory cytokines, such as IL-6, TNF, IL-1β, IL-2, IL-10 and C-reactive protein, in PD patients compared with healthy control individuals [16]. The disruption of the blood–brain barrier has been identified as a critical factor for PD [17]. This blood–brain barrier dysfunction could lead to the increased infiltration of peripheral immune cells (T and B lymphocytes) into the central nervous system [16]. In addition, IL-6, TNF and IL-1β could cross the blood–brain barrier and stimulate neuroinflammatory reactions under pathologic conditions [18]. In healthy adults and elderly people, physical activity is associated with lower levels of inflammatory markers. The exercise-induced anti-inflammatory effects have been reported to involve a differential cytokine response represented by decreased circulating levels of certain inflammatory biomarkers (IL-6, IL-1β and IFN-γ) and a suppression of TNF production [19]. The sustained modulation of the circulating levels of cytokines may influence brain function [20]. In a very recent study, the authors reported that this sustained modulation of the circulating levels of cytokines might play an important role in improving the motor function of PD patients after Tai Chi exercise [14]. However, the gene changes induced by Tai Chi that influence peripheral immunity among patients with Parkinson’s disease are still unknown. The specific molecular mechanisms of the peripheral immune response to Tai Chi training are also not fully understood. To address the above, we obtained the gene expression profile (GSE124676) for the peripheral leukocytes of PD patients before and after participating in Tai Chi exercise from the study of Hu et al. [21]. The study’s researchers used Tai Chi and multimodal exercise training to explore the responses in terms of shared differentially expressed genes (DEGs) and functional enrichment in the leukocytes of PD patients. In total, 1453 downregulated and 417 upregulated DEGs were obtained after analysis, and the DEGs were mainly enriched in the T-cell receptor signaling pathway, primary immunodeficiency pathway and osteoclast differentiation pathway. However, their study did not include data analysis to identify changes in the potential key candidate genes and explore important interactions between genes induced by Tai Chi exercise training. Weighted gene correlation network analysis (WGCNA) has been proven to be a powerful method for constructing co-expression networks based on gene expression data. Accordingly, the first aim of this study was to further identify the potential key candidate genes involved in responses to Tai Chi training, and a secondary aim was to explore the related regulatory mechanism of the peripheral immune changes in PD patients after Tai Chi interventions.

## 2. Materials and Methods

### 2.1. Microarray Data

In this study, the preprocessed gene expression profile in the fpkm form of GSE124676 was downloaded from the Gene Expression Omnibus (GEO) database (https://www.ncbi.nlm.nih.gov/geo/, accessed on 25 February 2022). The differentially expressed genes (DEGs) in the peripheral leukocytes of PD patients before and after Tai Chi exercise were identified and then analyzed by using WGCNA. In addition to the functional enrichment analysis, a protein–protein interaction (PPI) network was constructed. This dataset comprised 21 patients with PD who completed a 12-week Tai Chi training program. The patients’ characteristics (mean ± SD) were as follows: (1) age, 65.71 ± 11.85 years; (2) sex (*N*), 14/7 (male/female); (3) BMI (body mass index), 23.67 ± 2.82; (4) duration of disease, 4.98 ± 3.16 years; (5) MMSE (mini-mental state examination), 27.81 ± 2.32; and (6) levodopa equivalent doses, 360.20 ± 226.61 (mg/day). Blood samples were collected from them to analyze the molecular responses in the peripheral leukocytes before and after the Tai Chi exercise. Since the dataset was obtained from a public database, no ethical approval was needed for the present study.

### 2.2. Screening of DEGs

The DEG analysis among samples was performed utilizing the Wilcoxon rank-sum test [22], and the DEGs were screened with thresholds of *p* < 0.05 and |log fold change (FC)| > 2. The Wilcoxon rank-sum test was used to examine whether proteins from the peripheral leukocytes of PD patients were differentially expressed before and after Tai Chi exercise. Upregulated or downregulated proteins in peripheral leukocytes were defined as potential key candidate genes.

### 2.3. WGCNA for DEGs

The co-expression network was constructed using the WGCNA package in the R language [23]. We conducted a signed WGCNA based on GSE124676. The GSE124676 matrix that contained the gene expression was ranked by the standard deviation (SD), and the top 1500 genes were selected for the next WGCNA. The hclust function was used to perform cluster analysis on the samples and eliminate outlier samples in the dataset. The correlation between the two genes was calculated by introducing a weighted coefficient, and the adjacency matrix of the gene expression profile was calculated. The soft threshold β was reasonably selected within a certain range according to the scale-free network fitting index and average connectivity. In this study, the fitting index R^2^ > 0.85 was set to make the connection between genes that obeyed the approximate scale-free network. We set the minimum number of genes in each module to 20 and the cluster analysis height of the module to 0.2 in the identification of gene modules.

### 2.4. Functional Enrichment Analysis for the Genes in Key Modules 

Function enrichment pathway analysis for module genes was performed by using the “org.Hs.eg.db” and “clusterProfiler” packages in R [24]. Firstly, we used the “org.Hs.eg.db” package to transfer the gene symbols to entrezIDs for subsequent analysis. Then, the “clusterProfiler” package was utilized to find the enriched Kyoto Encyclopedia of Genes and Genomes (KEGG) and Gene Ontology (GO) items, where FDR < 0.05 (BH-corrected) was set for statistical significance. The minimal and maximal sizes of the genes annotated for testing were 10 and 500, respectively. Additionally, the background distribution by default was all the genes that had annotations.

### 2.5. Construction of Protein–Protein Interaction Network 

The genes in key modules were integrated and uploaded to the STRIN G database (version: 11.5; http://www.string-db.org/, accessed on 29 April 2022). The protein–protein interactions (PPIs) were retrieved with the following parameters: the species was set as human, and the edges indicated both functional and physical protein associations. To study the interactions among module genes, all the genes from the black module were searched and analyzed using the online Search Tool for the Retrieval of Interacting Genes (STRING database, Version 11.5; http://string-db.org/, (accessed on 30 April 2022)); then, the network was established through protein–protein interaction (PPI) analysis [25]. In our database search, we set the species to ‘Homo sapiens’, the confidence score cutoff at 0.4, the meaning of network edges as confidence with the line thickness indicating the strength of data support, and the other settings to default.

## 3. Results

### 3.1. DEG Screening 

**Figure 1:**
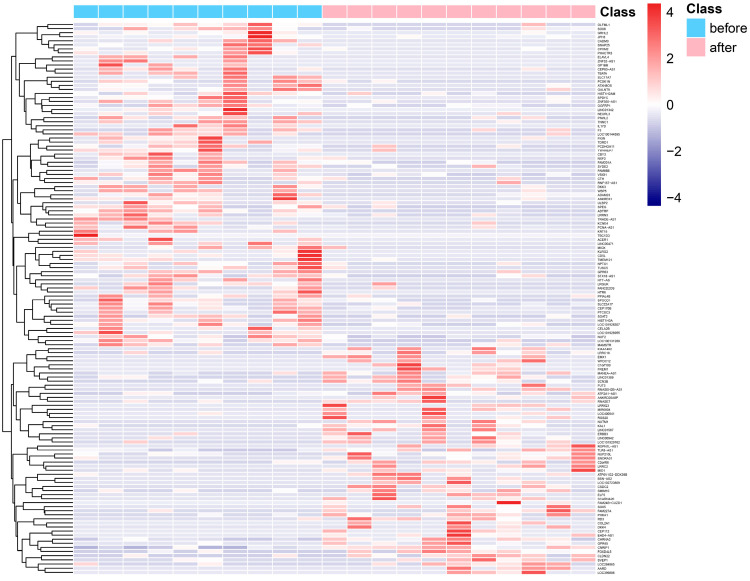
Heatmap shows differentially expressed genes (DEGs) before or after 12-week Tai Chi exercise; red represents highly expressed genes, and blue represents lowly expressed genes.

Figure 1 displays the hierarchical clustering and heatmap visualization of differentially expressed genes in peripheral leukocytes of PD patients before or after Tai Chi intervention. The X axis and the Y axis represent sample and genes, respectively. Class refers to the sample type, including samples before and after Tai Chi exercise. According to the screening criteria, a total of 136 DEGs were found in the Tai Chi participants (Figure 1). Among them, 56 genes including FREM1, RNASE7, MID1 and RGS20 were upregulated and 80 genes including OLFML1, GRHL2, JPH3 and CADM3 were downregulated after Tai Chi training. RNASE7 is a public differentially expressed gene that was in a previously reported set of DEGs in Parkinson’s patients’ skin [26]. RNASE7 was found to show lower expression in the skin in PD. As one of the 50 most significantly changed genes in PD versus healthy subjects, RNASE7 is the antimicrobial peptide showing the greatest potential and a key effector molecule of the innate immune system. In addition to being expressed in various epithelial cells (e.g., skin and respiratory tract cells), it is also contained in or secreted by leukocytes and platelets as part of an intravascular defense system [27]. Interestingly, our analysis found that the expression of RNASE7 in the blood leukocytes of PD patients was upregulated after Tai Chi exercise, indicating that Tai Chi may be involved in regulating the abnormal immunity in patients with PD. 

### 3.2. WGCNA 

**Figure 2:**
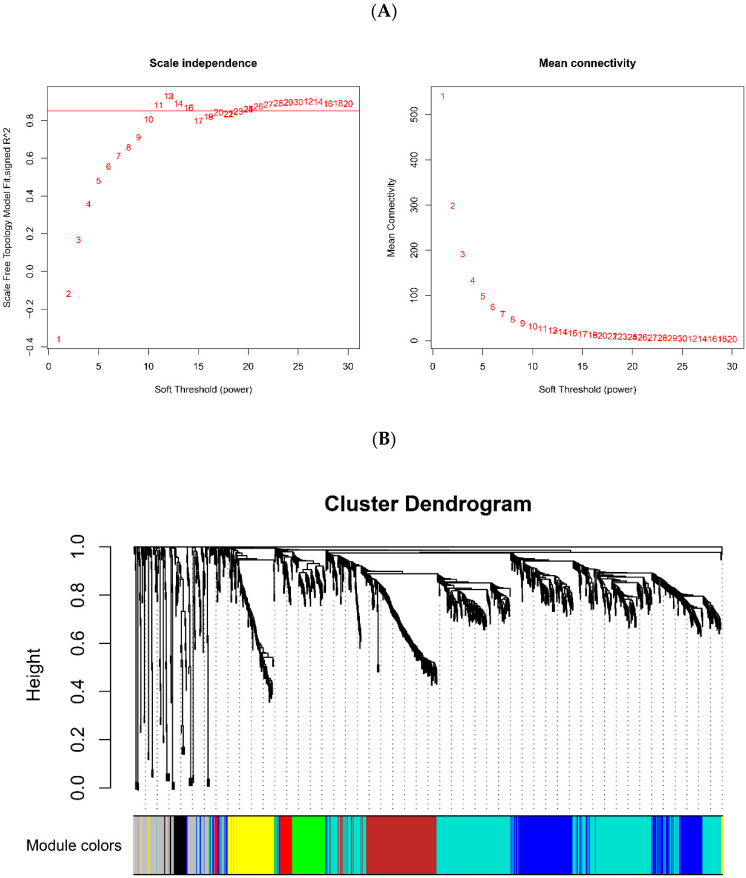

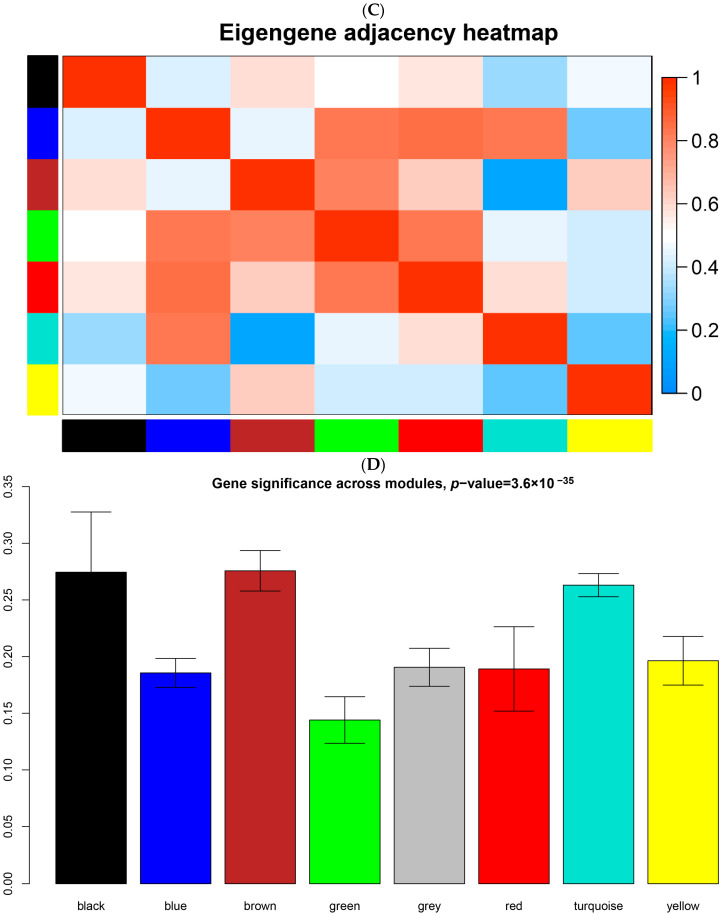
Weight gene co-expression network analysis. (**A**) Scale independence and mean connectivity analysis. (**B**) Cluster dendrogram and module partitioning of the co-expression gene modules. The 8 colors indicate the corresponding gene co-expression modules. (**C**) Heatmap for module eigengenes. The correlation of module eigengenes increases gradually with color changing from blue to red. (**D**) Significant modules associated with peripheral immunity in PD patients before and after Tai Chi intervention.

We selected the top 1500 genes ranked by the standard deviation (SD) and performed WGCNA. According to the scale-free network fitting index and average connectivity, β = 11 was calculated and selected as the soft threshold of this dataset (Figure 2A). The adjacency matrix was converted into TOM based on the topological overlap. The clustering method was used to cluster genes with high topological overlap to construct a clustering dendrogram. The dynamic tree cut method [28] was used to prune and perform further classification of the modules. In this manner, the genes with high topological overlap were clustered into the same module. Thereafter, the genes were divided into eight modules, which were represented by eight color rectangles, respectively. The ordinate was the proportion of genes. The branches of the dendrogram correspond to eight different gene modules. Each leaf on the dendrogram corresponded to a gene. The genes were clustered into modules of the same color (Figure 2B). A heatmap of the module eigengenes was plotted according to the significance of the correlations between each gene module, as shown in Figure 2C. It can be seen in Figure 2C that different color blocks (mainly red and blue) in the module gene map have different distribution regions, implying that the module eigengenes were not highly correlated with each other in different modules. In addition, there was no significant co-expression correlation between the module characteristic genomes of the different modules, indicating that the different modules had strong independence. The results showed that the modules were worth our doing further analyses and research. [29]. Hierarchical clustering and heatmap analysis were performed on each module. To identify the significance of each module, we calculated the gene significance (GS) to measure the correlation between genes and sample traits before or after Tai Chi exercise. The module significance (MS) was defined as the average GS of the genes within the module and was calculated to measure the correlation between modules and sample traits. The association of co-expression modules with sample traits was calculated using Pearson’s correlation tests, and the results were plotted in barplot or boxplot form. Each bar or box was labeled with the corresponding module color. The correlation between the modules showed that three modules (Black, Brown and Turquoise) were distributed in different subtrees of the clustering tree. The above three modules had high correlations with the changed genes in peripheral leukocytes before and after Tai Chi exercise (Figure 2D). Since these data are more representative, it is reasonable for us to focus on these three modules in the subsequent analyses.

### 3.3. Expression Levels of the Genes in Key Modules

**Figure 3:**
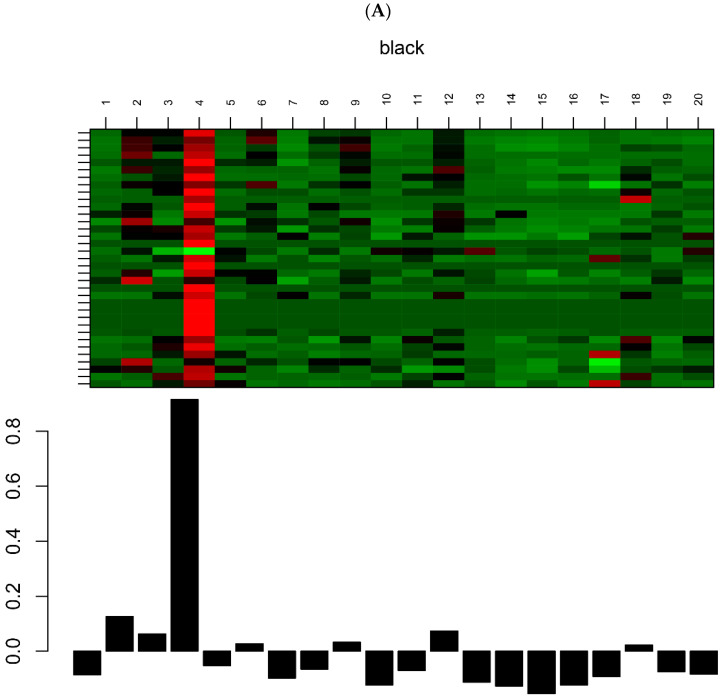

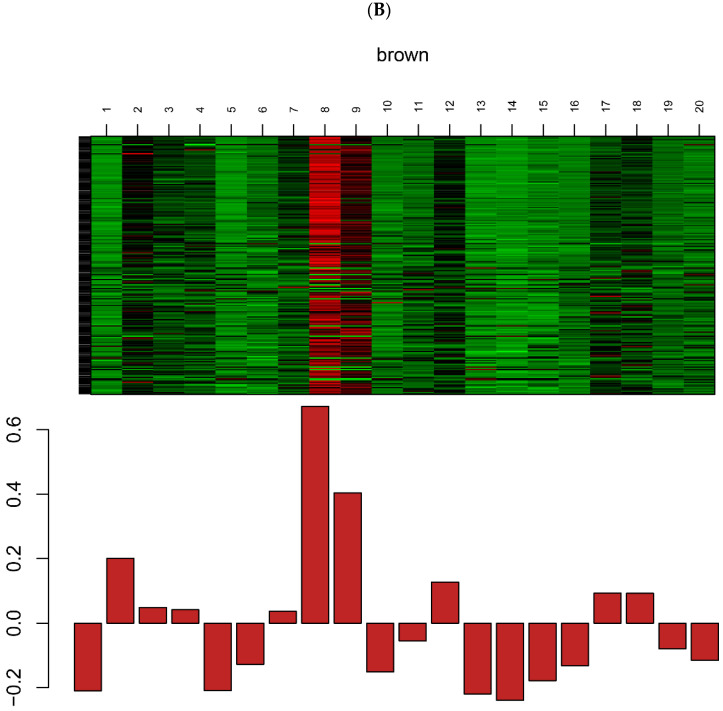

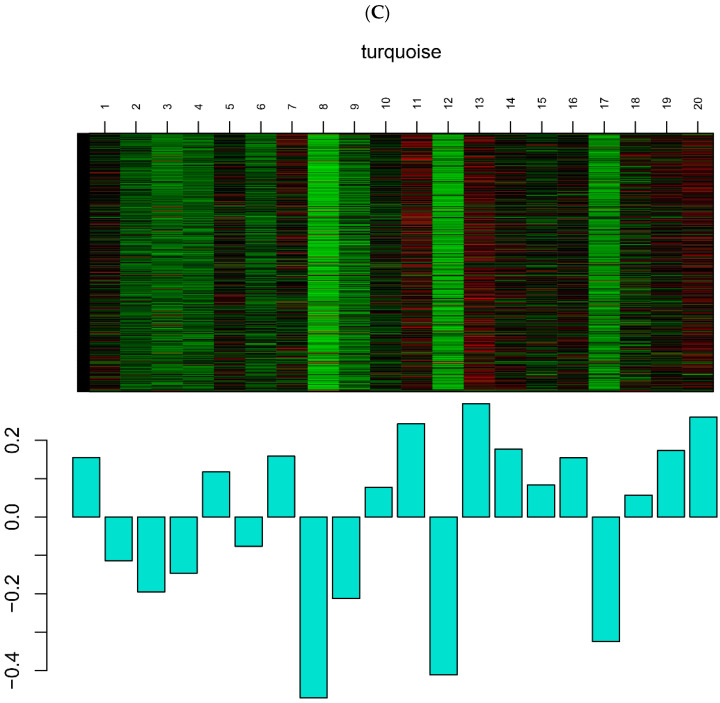
The heatmap and histogram of gene expression patterns in the Black (**A**), Brown (**B**) and Turquoise (**C**) modules.

Hierarchical clustering analysis was performed with the flashClust function and the results were presented in Figure 3A–C. We found one obvious outlier (GSM3539269) which was removed from the cohort. Thus, the gene expression values of the other 20 samples were adopted to construct co-expression modules using the WGCNA algorithms (Supplementary Materials Figure S1). In total, three significant key modules (Black, Brown and Turquoise) were identified after WGCNA. There were 35, 201 and 540 genes to be included in the Black, Brown and Turquoise modules, respectively. Figure 3A–C represents a visualization of the gene expression levels and the eigengene values of the three modules. The clustering heatmap and eigengene bar plot display the gene expression levels of each module. The heatmap shows a red and green color image of the gene expression. Larger and smaller entries are represented with reds and greens of increasing expression, respectively. The bar plot displays the eigengene expression of the corresponding module, with the X and Y axes representing the sample and eigengene expression, respectively. Since the module eigengene (first principal component) represents a suitably defined average gene expression profile, it can be seen that the eigengene expression is highly correlated with expression of genes in the whole module. 

### 3.4. Functional Enrichment Analysis for the Genes in Key Modules

**Figure 4:**
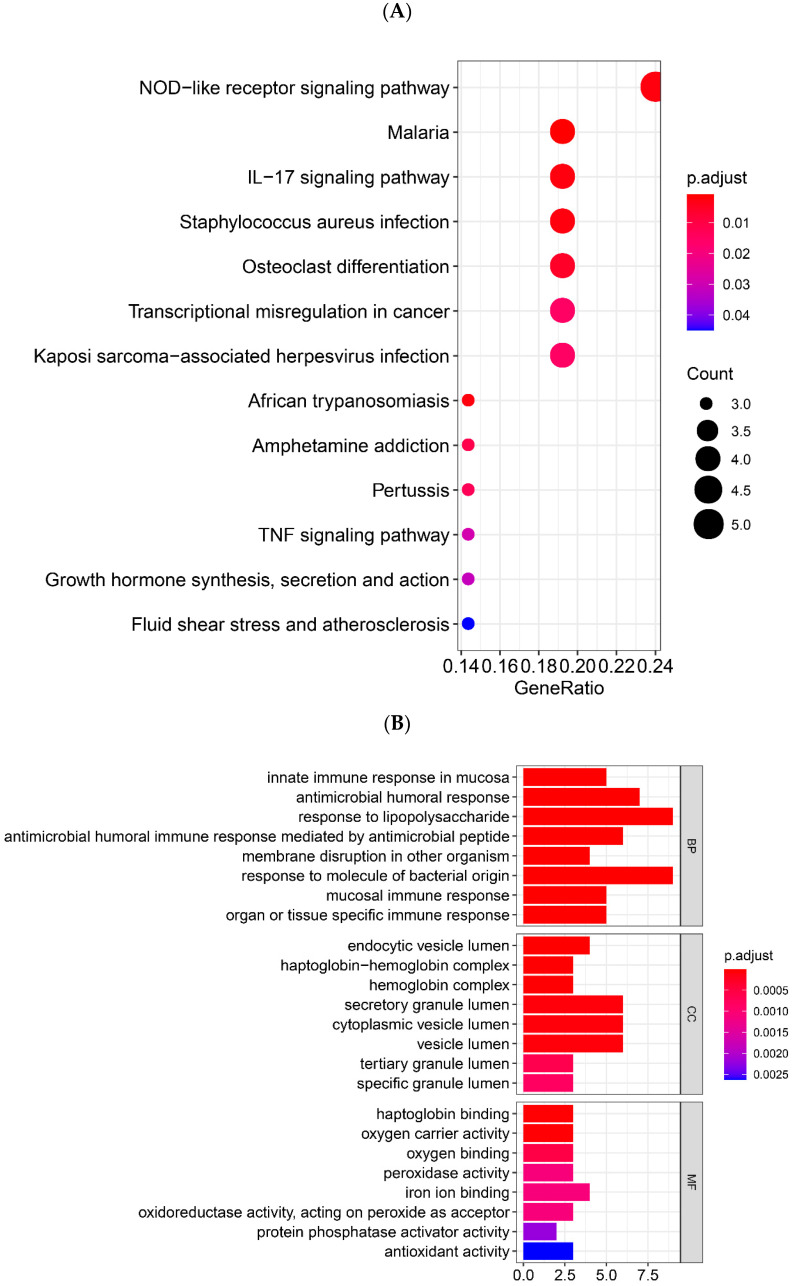
GO and KEGG enrichment analysis of black modules. (**A**) KEGG pathway analysis of black modules. (**B**) GO function enrichment analysis of black module.

Based on the WGCNA results, the DEGs in the Black module showed the strongest correlation with the Tai Chi intervention. Therefore, the function enrichment analysis results for the Black module were highlighted and further analyzed. GO and KEGG pathway enrichment analysis was performed to elucidate the biological functions and pathways of genes and pathways, respectively (Figure 4A,B). In terms of biological processes, the genes in the Black module are mainly involved in responses such as lipopolysaccharide, molecule of bacterial origin, and human antibacterial response process. In terms of molecular function, these genes are mainly enriched in haptoglobin binding, oxygen carrier activity, and oxygen binding process, etc. In cellular components, secretory granule lumen, cytoplasmic vesicle lumen, and vesicle lumen are the most significant enrichment terms. Moreover, the genes participate in malaria, NOD-like receptor signaling pathway, and IL-17 signaling pathways. The genes in the Brown module are primarily enriched for viral gene expression and translational initiation (Supplementary Materials Figure S2). The genes in the Turquoise module are mainly enriched for neutrophil activation and T cell activation (Supplementary Materials Figure S3). Two studies on the transcriptomic profiles of PD patients’ skin and blood found that the enriched pathways were mainly in Huntington disease, non-alcoholic fatty liver disease (NAFLD), Parkinson disease and cholesterol metabolism [26,30]. Our study mainly showed enrichment in malaria, NOD-like receptor signaling pathway, and IL-17 signaling pathways. It can be speculated that the impact of Tai Chi on the PD peripheral immune response involves the NOD-like receptor and IL-17 signaling pathway, which may be beneficial for developing new ideas for the treatment of PD.

### 3.5. Construction of PPI Network 

**Figure 5:**
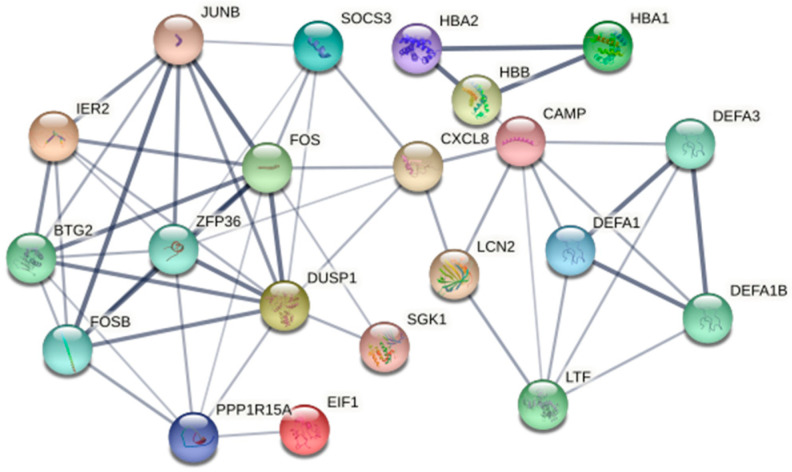
The 21 hub genes in the Black module among the PPI network.

It is known that regular exercise exerts many health benefits, which may partly involve the regulation of inflammation by the Nod-like receptor signaling pathway [31], IL-17 signaling pathway [32] and TNF signaling pathway [33]. However, these exercise-related signaling pathways were not found in the Brown and Turquoise modules after Tai Chi training. Therefore, the Black module further served as a key module for hub gene screening and analysis of the interaction relationship between the genes. After removing the non-gene codes, 21 hub genes were screened in the Black module, including FOS, FOSB, ZFP36, CAMP, JUNB, LCN2, IER2, BTG2, PPP1R15A, EIF1, SGK1, DUSP1, SOCS3, CXCL8, LTF, DEFA1B, DEFA1, DEFA3, HBB, HNA1 and HBA2 (Figure 5).

## 4. Discussion

Tai Chi has been considered one of the most promising exercise programs for improving human dysfunction in neurological disorders [34]. An anti-inflammatory response in Tai Chi exercise studies was identified as indicated by either decreased proinflammatory cytokines (IL-1, IL-2, IL-8, IL-12, IFN-γ and NF-kβ) or increased anti-inflammatory cytokines (IL-13) [35]. Proinflammatory responses are involved in the development of neuroinflammation and neurodegenerative pathology in PD [36]. A recent study has reported that Tai Chi could improve motor function by altering the peripheral inflammatory environment in PD patients [14]. However, the relevant molecular mechanisms of Tai Chi exercise and the changes in peripheral immunity in PD patients remain to be determined and elucidated. Thus, in this study, we examined whether changes occurred in potential key candidate genes for peripheral immunity following Tai Chi among patients with Parkinson’s disease. In the following analysis, we also explored the possible mechanism of Tai Chi exercise’s impact on peripheral immunity in PD patients. In the present study, WGCNA was performed on an RNA-seq dataset downloaded from the GEO database, and the genes differentially expressed in the peripheral leukocytes of PD patients before and after Tai Chi exercise were calculated. WGCNA can construct co-expression networks based on expression data and is increasingly being used in biology to analyze large, high-dimensional datasets. Using module eigengenes, WGCNA can reconstruct gene co-expression modules and summarize modules. WGCNA can avoid the limitations of research on datasets based only on traditional differential gene-screening methods [21], which may not allow core analysis of the regulatory process and make it difficult to explore the overall biological system. Based on this method, this study identified differentially expressed genes, used the found gene-interaction information to identify gene modules related to Tai Chi, and obtained key genes through the construction of a PPI network.

The top 1500 genes were selected for a WGCNA. In the WGCNA, eight color modules were identified and clustered, and correlation analysis between the genes and peripheral immunity phenotypes was carried out for each module. Two key findings emerged from the present study. First, among the eight modules, the Black module is the most correlated with Tai Chi exercise and, therefore, it may be the most significant influential module. Second, the 21 hub genes in the Black module were observed and six key candidate genes (FOS, FOSB, JUNB, ZFP36, CAMP and LCN2) were identified as the significant influential genes possibly eliciting positive changes in peripheral immunity induced by Tai Chi training in PD individuals.

In the Black module, the gene functions were found to be mainly enriched in biological processes such as response to molecule of bacterial origin, organ or tissue specific immune response, innate immune response in mucosa and antimicrobial humoral response. In addition, the KEGG pathway analysis showed that the differentially expressed genes were involved in NOD-like receptor signaling pathway, IL-17 signaling pathway, and TNF signaling pathway. The Nod-like receptors are a family of pattern recognition receptors that are involved in identifying pathogen-associated molecular patterns and damage-/danger-associated molecular patterns [37]. The Nod-like receptors are divided into four subfamilies. The NLRP subfamily is mainly involved in the development of the inflammasome complex and is the one most closely related to exercise [31]. The chronic activation of microglia causes an NLRP3-inflammasome-mediated release of potent proinflammatory cytokines—interleukin (IL)-1β and IL-18—which are thought to be responsible for neurodegeneration [38]. Long-term moderate-intensity exercise was reported to reduce the expression of the NLRP3 gene and serum IL-1β and IL-18 [39]. Reduced NLRP3 expression in peripheral blood mononuclear cells (PBMCs) [40] was also found with resistance training. All these suggest that NLRP3-inflammasome activation may be prevented by exercise. IL-17 is classified as a proinflammatory cytokine, and signaling by IL-17 increases matrix metalloproteinase and proinflammatory cytokine expression [41]. Neutrophil activation and muscle damage during and after prolonged endurance exercise are associated with increased IL-17 [32]. TNF-α, an inflammatory cytokine, is commonly found to be elevated in PD and may play an important role in PD symptoms and progression [42]. A previous pilot study indicated that Qigong exercise might reduce the serum level of TNF-α in people with PD [43]. The common point of these three main pathways is participation in the progression of PD by regulating inflammation. However, the inflammation may be affected by different exercise modes through these three pathways. This may also be one of the mechanisms by which Tai Chi exercise affects peripheral inflammation in PD.

By the integration of hub genes from the Black module and DEG analysis, we identified 21 potential key candidate genes including FOS, FOSB, JUNB, ZFP36, CAMP and LCN2 in the Black module. FOS is a nuclear phosphoprotein encoded by the mature mRNA transcribed from the c-FOS gene [44]. It is an inherent gene in human or mammalian cells, also known as an immediate early response gene. c-FOS is an immediate early gene widely present in the central nervous system. Its encoded c-Fos protein is often used to characterize the activity of neurons [45]. Previous studies have reported a decrease in c-FOS in the subthalamic nuclei of Parkinson’s patients [46]. The expression of FOS, c-FOS and JUNB in the skeletal muscle was significantly upregulated after acute exercise [47], while the c-FOS and c-JUN mRNA significantly increased in rats’ hippocampi after 8-week swimming training [48]. However, there is no report on the effect of exercise on FOS, FOSB and JUNB in the peripheral blood. To our knowledge, this is the first study to observe that FOS, FOSB and JUNB are potential key candidate genes in the effect of Tai Chi exercise on peripheral immunity in PD patients. As a transcription factor, the FOS protein is an important regulator of cell growth, division, proliferation, differentiation and even programmed death [49]. The FOS protein and its family members (FOSBs) can bind to the c-Jun/JUNB protein to form a dimer, AP-1, which is an active transcription factor [50]. Zhao et al. reported that AP-1 transcription and translation were significantly increased in MPTP-induced PD mice, and they speculated that AP-1 played a key role in neurodegeneration following brain injury, potentially via its involvement in inflammatory processes [51]. Accordingly, we suggest that the FOS, FOSB and JUNB proteins can activate AP-1 and promote the positive changes in peripheral inflammation after Tai Chi exercise.

ZFP36 (Tristetraprolin, TTP) is one of the most well-studied transcription factors. It destroys target mRNAs by binding to adenosine uridine (AU)-rich elements in the 3′-untranslated regions and recruits deadenylation and degradation factors. ZFP36 has been known to function as an anti-inflammatory modulator in murine models of systemic inflammatory diseases by downregulating the production of various proinflammatory cytokines, including tumor necrosis factor α (TNF-α) [52]. Furthermore, ZFP36 can also influence inflammation through binding to p65, attenuating NF-κB nuclear translocation, and thus preventing the transcriptional activation of NF-κB target genes, including those encoding proinflammatory cytokines [53]. It was found that the nicotinergic stimulation of skeletal myotubes, mimicking the activation of the motor endplate by neuronal signals, led to increased ZFP36 levels. This would result in reduced inflammation, as reflected by a decrease in the production of proinflammatory cytokines [54]. All these indicate that ZFP36 may play an important role in inflammation and repair and is possibly involved in multiple signaling cascades of skeletal muscle homeostasis. 

CAMP (cyclic adenosine monophosphate, cAMP) is the first discovered intracellular second messenger generated by ATP under the action of adenosyl cyclase. It plays a key role in muscle contraction, neurotransmission, vision, differentiation, cell growth and secretion [55]. PKA is a key regulator of cAMP and is involved in synaptic plasticity and the formation of long-term memory. cAMP can activate PKA by binding to regulatory subunits. The cAMP/PKA-related pathway and the downstream cell signaling cascades can enhance the function of dopaminergic neurons, improve dopamine signaling, increase the release of neurotrophic factors and reduce the expression of α-synuclein. All these enhanced functions could contribute to positively changing the pathological process of Parkinson’s disease [56]. This suggests that Tai Chi training may provide such benefits as an effective therapy for PD patients.

LCN2(Lipocalin 2, Lcn2) is a member of the highly heterogeneous secreted protein family of lipocalins but has diverse physiological roles such as the regulation of the innate immune response [57], cellular iron transport [58] and neuroinflammation [59]. Under normal physiological conditions, LCN2 expression is rather low in the brain, but it is remarkably upregulated under pathological conditions such as inflammation or injury [60]. Kim et al. identified increased LCN2 expression in the substantia nigra (SN) of patients with PD [61]. LCN2 may be a key trigger of neuroinflammation, by promoting the production of neurotoxic proinflammatory cytokines such as TNF-α and IL-1β in the central nervous system, leading to the further disruption of nigrostriatal DA projections. In addition, Edison et al. found that serum LCN2 levels were significantly higher in PD patients than in healthy controls [62]. Thus, changes in the activity or level of LCN2 induced by Tai Chi training may modulate or alter its diverse physiological roles in the peripheral immune systems of PD patients.

The remaining fifteen key candidate genes (IER2, BTG2, PPP1R15A, EIF1, SGK1, DUSP1, SOCS3, CXCL8, LTF, DEFA1B, DEFA1, DEFA3, HBB, HNA1 and HBA2) have not been found to be significantly associated with PD and exercise. The findings of this study should be interpreted in light of its limitations. First, due to the small sample size of the GSE124676 dataset, it cannot fully represent PD patients. Caution should be taken when generalizing the results of the present study. Secondly, the correlation between some key candidate genes and PD has not been verified by biological experiments. Thus, more and larger-scale clinical investigations using Tai Chi interventions are needed to verify these findings, particularly the relationships between the key genes, PD and Tai Chi, to judge whether they can be used as new targets.

In summary, the use of WGCNA in the current study allowed us to identify potential gene modules including 21 key genes in the Black module. These key genes were strongly correlated with Tai Chi training, which could potentially affect peripheral immunity in PD patients. The relevance of the possible mechanism is supported by the fact that Tai Chi exercise training interventions in the PD individuals induced six key candidate genes (FOS, FOSB, JUNB, ZFP36, CAMP and LCN2). They are significant influential genes that could possibly elicit positive changes in peripheral immunity. It will be clinically meaningful to verify our findings by applying Tai Chi interventions to a larger cohort of healthy volunteers, and to also include a larger number of patients with PD. Our work suggests that there is possibly great therapeutic potential in targeting these identified hub genes—FOS, FOSB, JUNB, ZFP36, CAMP and LCN2—in PD patients, thus harnessing one of the many beneficial effects of Tai Chi training after being confirmed by large but sound clinical trials.

## 5. Conclusions

In conclusion, this study used the WGCNA to identify changes of peripheral immune biomarkers response to Tai Chi exercise in PD patients. We observed six key candidate genes (FOS, FOSB, JUNB, ZFP36, CAMP and LCN2) that were involved in peripheral inflammation and the inhibition of in-flammation induced by Tai Chi training. These genes could be potentially used as targets for future research on molecular mechanisms of the Tai Chi influence on peripheral inflammation in PD patients.

## Data Availability

All data generated or analyzed during this study are included in this published article.

## Supplementary Materials

The following supporting information can be downloaded at: https://www.mdpi.com/article/10.3390/genes13101863/s1, Figure S1: Sample clustering; Figure S2: Go and KEGG enrichment analysis of brown modules. A: GO pathway analysis of brown modules. B: KEGG function enrichment analysis of brown module; Figure S3: Go and KEGG enrichment analysis of turquoise modules. A: GO pathway analysis of turquoise modules. B: KEGG function enrichment analysis of turquoise module.
