# Supplementary material for: Changes Observed in Potential Key Candidate Genes of Peripheral Immunity Induced by Tai Chi among Patients with Parkinson’s Disease
Source: Genes (Basel). 2022 Oct 15;13(10):1863. doi: 10.3390/genes13101863 (PMC9601924; doi:10.3390/genes13101863)
Supplement: Supplementary file 1 [file genes-13-01863-s001.zip › genes-1878004-supplementary.pdf]

Changes observed in potential key candidate genes of peripheral immunity induced by Tai Chi among patients with Parkinson's disease

Guang Yang[1*], Qun Dong[2*], Huixin Yang[3], Fan Wang[3], Linwei Chen[3], Junze Tang[3], Guoyuan Huang[4#] and Ying Zhao[1#]

[1] Departmen of Physical Education t, Shanghai Jiao Tong University, Shanghai 200240, China

[2] Department of Bioinformatics and Biostatistics, School of Life Sciences and Biotechnology, Shanghai Jiao Tong University, Shanghai, 200240, China.

[3] Institute of Nation Traditional Sports, Harbin Sport University, Harbin, 150006, China

[4] Pott College of Science, Engineering and Education, University of Southern Indiana, Indiana 47712, USA

* Co-first-authors contributed equally to this work.

# Correspondence: ghuang@usi.edu; zhaoying514@163.com

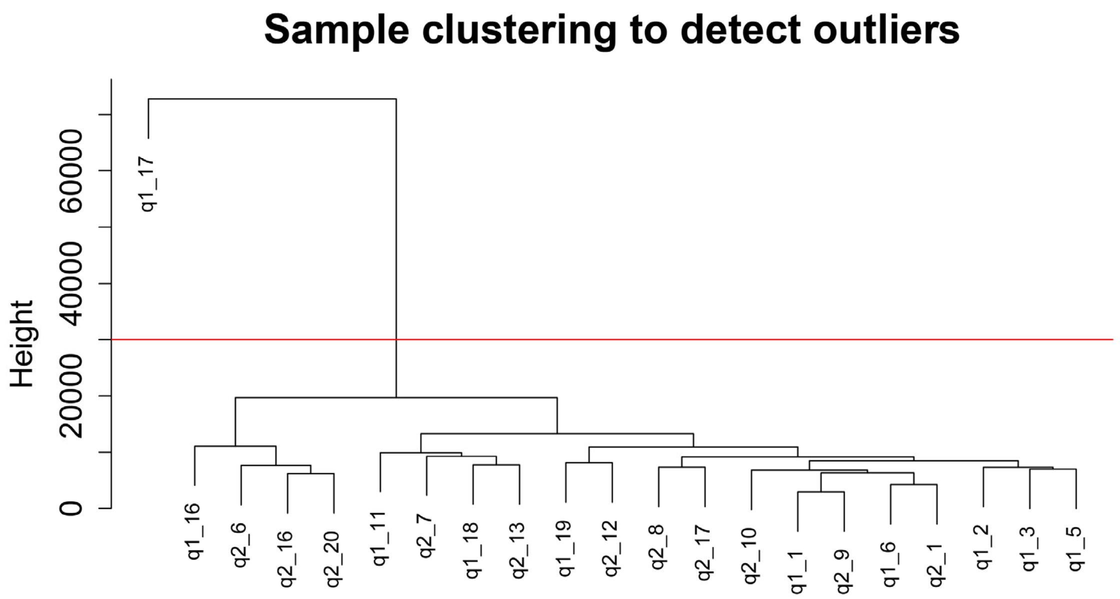

Figure S1. Sample clustering.

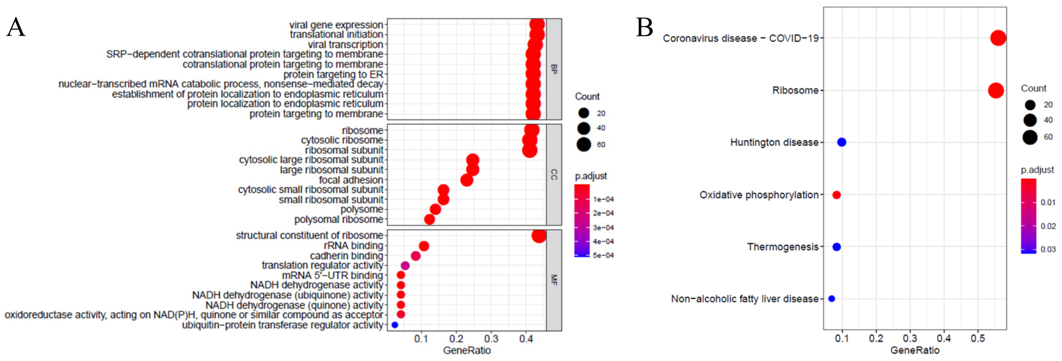

Figure S2. Go and KEGG enrichment analysis of brown modules. A: GO pathway analysis of brown modules. B: KEGG function enrichment analysis of brown module.

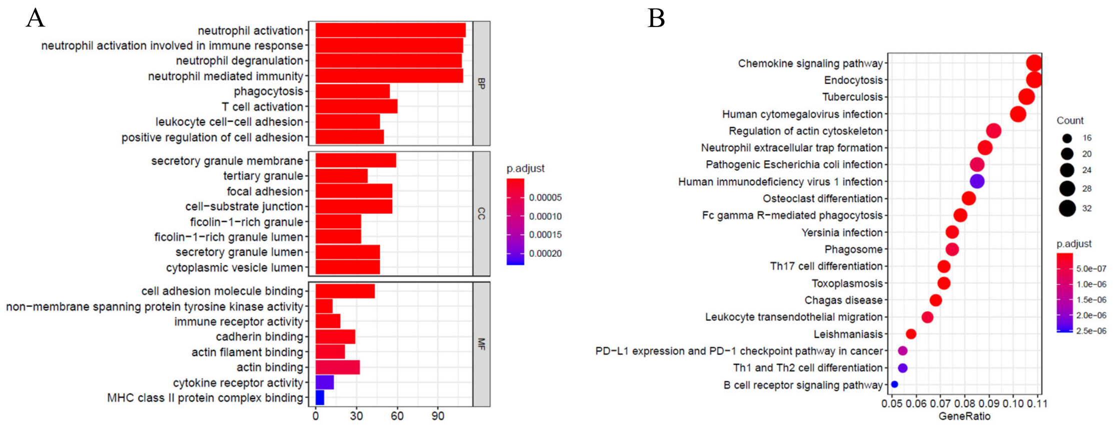

Figure S3. Go and KEGG enrichment analysis of turquoise modules. A: GO pathway analysis of turquoise modules. B: KEGG function enrichment analysis of turquoise module.
